# Case Report of *Rhodococcus erythropolis* Contamination in Buruli Ulcer Lesions: Effects on *Mycobacterium ulcerans* Diagnosis

**DOI:** 10.1155/crdi/6627150

**Published:** 2026-06-01

**Authors:** Francis Zeukeng, Jennifer Seyram Amedior, Evelyne Fegue Ndzodo, Solange E. Kakou-Ngazoa, Stephen Mbigha Ghogomu, David N’golo Coulibaly, Wilfred Fon Mbacham, Jude Daiga Bigoga, Anthony Ablordey

**Affiliations:** ^1^ The Biotechnology Centre (BTC), University of Yaoundé I, P.O. Box 17673 Etetak, Yaoundé, Cameroon, uy1.uninet.cm; ^2^ Department of Biochemistry and Molecular Biology, Faculty of Science, University of Buea, P.O. Box 63, Buea, Cameroon, ubuea.cm; ^3^ Department of Bacteriology, Noguchi Memorial Institute for Medical Research, University of Ghana, P.O. Box 581, Legon, Accra, Ghana, ug.edu.gh; ^4^ Department of Technics and Technology, Platform of Molecular Biology, Pasteur Institute Abidjan, P.O. Box 490 Abidjan 01, Abidjan, Côte d’Ivoire, pasteur.ci

**Keywords:** case report, *Mycobacterium erythropolis*, *Mycobacterium ulcerans* infection, *Rhodococcus erythropolis*, skin NTDs, whole-genome analysis

## Abstract

**Introduction:**

We describe cases of contamination of *Mycobacterium ulcerans* infections (Buruli ulcer) with *Rhodococcus erythropolis*, a bacterium of environmental origin that is rarely associated with human infection.

**Case Presentation:**

The infectious pathogen of Buruli ulcer, *Mycobacterium ulcerans*, was detected and cultured in vitro from two lesion swabs taken from clinically described Buruli ulcer–like patients (4 and 34 years old). Infection by *M. ulcerans* was confirmed using the WHO‐recommended IS2404/IPC‐qPCR multiplex analysis of DNA extracts from patient samples, which were subsequently categorized as positive. PCR‐positive samples were incubated in Löwenstein–Jensen (LJ) culture media, and colony phenotypes characteristic of *M. ulcerans* were observed 14 days postincubation. However, analysis of culture suspensions by qPCR revealed no *M. ulcerans* DNA, while Ziehl–Neelsen (ZN) staining showed phenotypes closely related to acid‐fast bacilli (AFB) of *M. ulcerans*. Detected AFBs were not clustered after ZN staining. Further microbial identification and characterization by MALDI‐TOF MS and Gram staining revealed the presence of *Rhodococcus erythropolis*. The identification was confirmed by whole‐genome sequencing (WGS) to establish the genomic link between this originally called *Mycobacterium erythropolis* and *M. ulcerans*. Analysis of short reads from WGS confirmed the organism as *R. erythropolis*. When employing the *M. ulcerans* Agy99 reference chromosome, comparative analysis of whole‐genome sequences revealed little genomic relatedness between the two organisms, with an average genome coverage of 5.72%.

**Conclusion:**

The study reports the first contamination cases of *M. ulcerans*–infected lesions with *R. erythropolis*. Although *R. erythropolis* did not interfere in the detection specificity of *M. ulcerans* by IS2404/IPC‐qPCR, it completely inhibited *M. ulcerans* growth in recommended LJ culture media, complicating routine biological diagnosis by culture and microscopy. Hence, Buruli ulcer is likely to be underdiagnosed due to lesion contamination by *R. erythropolis* and difficulties in *M. ulcerans* identification in the routine clinical diagnosis procedure.

## 1. Introduction

The study describes a case of confirmed *Mycobacterium ulcerans*–infected lesions (Buruli ulcer cases) with *Rhodococcus erythropolis*. The genus *Rhodococcus* refers to aerobic and Gram‐positive actinomycetes [1]. Species of this family possess a great metabolic ability, as they can degrade a range of environmental pollutants and transform or synthesize biocatalyst compounds with possible industrially useful applications. Although *Rhodococcus* species are globally considered to have low pathogenicity, they can cause diseases in animals, plants, and humans [2]. *R. erythropolis* is a bacterium of environmental origin with rare cases of human infection. It was originally considered a *Mycobacterium* species owing to its nonmotile and mycolate‐containing properties [3]. Clinically characterized cases of *R. erythropolis* infections have been described in humans from conjunctival samples, sputum (from a patient with pneumonia), and bloodstream [4–6], as well as in an immunocompromised patient with HIV and a patient treated with peritoneal dialysis [7, 8]. No other studies have reported its presence on other human secretions or sites, such as the skin surface necrotized by *M. ulcerans*. Our report adds *R. erythropolis* to the list of opportunistic human infectious pathogens of the skin. It can colonize *M. ulcerans*–infected active skin lesions, although its pathogenicity effect on skin invasion and disease severity has not yet been demonstrated. Additionally, the exact sources of contamination remain elusive but are presumed to be primarily from aquatic or soil environmental exposure facilitated by open wounds caused by the immunosuppressive effects of mycolactone, a Buruli ulcer virulence factor elaborated by *M. ulcerans*. As this species is likely to be a very rare cause of human disease, the details regarding diagnosis from this report can be used to inform patient management decisions in future cases, especially during colonization with *M. ulcerans* in Buruli ulcer patients. The report further draws a low genomic relatedness between the fast‐growing bacterium *R. erythropolis* and the slow‐growing germ *M. ulcerans*.

## 2. Case Report

**FIGURE 1:**
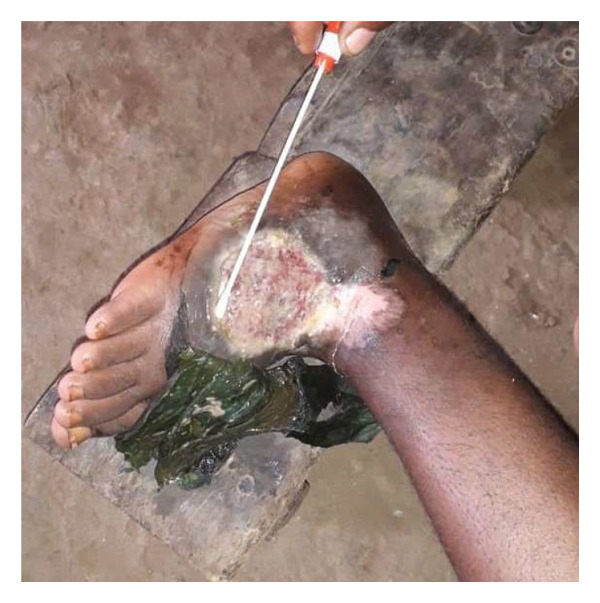
*Mycobacterium ulcerans*–infected wounds found with viable bacilli of *R. erythropolis*. This patient uses plant flowers from traditional healers to cover the wound.

A routine cross‐sectional sampling of lesion swabs from clinically suspected Buruli ulcer patients was conducted in August 2020 in Akonolinga health district, the primary Buruli ulcer endemic site in the Centre Region of Cameroon. The characteristics of this site have been published previously, as well as Buruli ulcer main risk factors that primarily include humid areas and several environmental materials [9, 10]. Buruli ulcer is known to be associated with long‐term disability and an enormous socioeconomic impact that considerably reduces patient hospitalization rates in endemic areas. Sample collection was achieved within the communities in local health facilities (district hospital, integrated health center, and district medical center), as well as in patient and traditional healers’ households. A total of 30 samples (lesion swabs and tissue biopsies) were collected from 30 Buruli ulcer–like wounds or skin injuries and submitted to routine microbial diagnostic methods following WHO guidelines [11]. Samples were collected in duplicate using sterile swab sticks (Figure 1) and suspended in screw‐cap tubes containing peptone water. Then, a composite sample was constituted in the laboratory for biological and molecular microbial analyses.

The primary usage of these samples was to generate baseline information on the genomic composition of *Mycobacterium ulcerans* (Buruli ulcer etiological agent) isolates circulating among Buruli ulcer patients in the study site. Collected samples were submitted to in vitro culture and whole‐genome sequencing (WGS) of *M. ulcerans*. Prior to culture, all 30 sample pools were subjected to decontamination with the mild oxalic acid method to reduce contamination by fast‐growing bacteria and fungi and increase recovery of *M. ulcerans* in culture. A portion of decontaminated samples was subjected to total genomic DNA extraction (DNasy Blood and Tissue Kit, Qiagen) and tested for detection of *M. ulcerans* DNA by IS2404/IPC multiplex real‐time PCR [12]. Another portion of the sample was subjected to Ziehl–Neelsen (ZN) staining to detect acid‐fast bacilli (AFB) by microscopy and cultured on Löwenstein–Jensen (LJ) solid medium at 33°C to recover *M. ulcerans* isolates [11].

**FIGURE 2:**
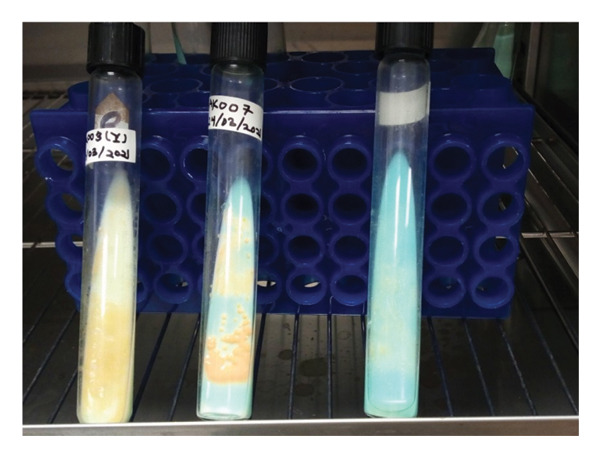
Colonies of *R. erythropolis* appear yellowish after 14 days of incubation at 33°C in Löwenstein–Jensen (LJ) culture media.

Detection by qPCR revealed the presence of *M. ulcerans* in 43.3% (13/30) of sample pools. All PCR‐positive and randomly selected negative samples were incubated on LJ at 33°C and monitored weekly for 6 months to trace *M. ulcerans* growth following the WHO guidelines [11]. After 2 weeks of incubation, microbial growth with yellowish colonies (Figure 2) was observed on two culture tubes that initially tested positive for *M. ulcerans* by qPCR. These fast‐growing colonies were less likely to be related to *M. ulcerans*, a slow‐growing mycobacterium with positive colonies seen on LJ media only after three‐four months of incubation at 29°C–33°C [11].

**FIGURE 3:**
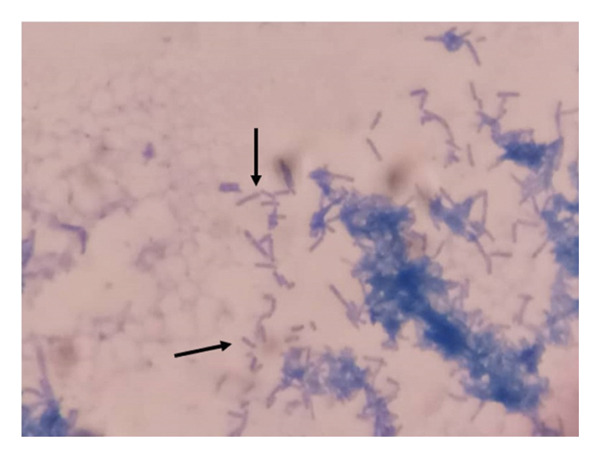
Acid‐fast bacilli (AFB) of *R. erythropolis* are not clustered after Ziehl–Neelsen staining. The black arrow points to AFB.

The observed yellowish phenotype on LJ media appeared to be very close to *M. ulcerans* colonies. Each suspicious colony was tested by ZN staining and qPCR to characterize *M. ulcerans*. Therefore, qPCR analysis of the two culture suspensions revealed the absence of *M. ulcerans* DNA, while ZN staining showed AFB (Figure 3).

**FIGURE 4:**
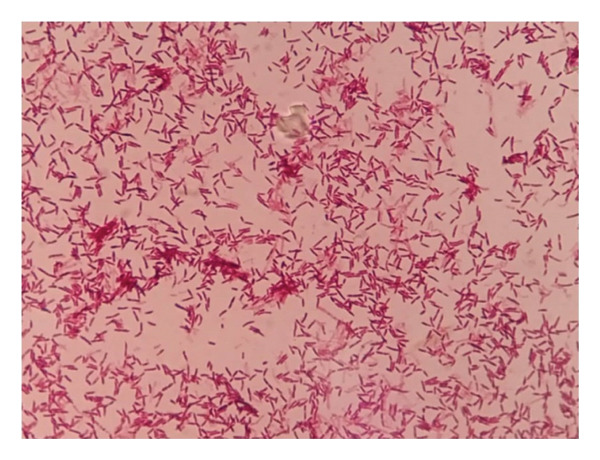
Gam staining revealing red bacilli of *R. erythropolis* (a Gram + bacterium) isolated from a Buruli ulcer–infected lesion.

In contrast to the AFB of *M. ulcerans*, which usually appears in clusters or clumps after ZN staining and sometimes forms cords or branching filaments, positive ZN strains of *R. erythropolis* appeared not clustered. Further microbial identification and characterization of these suspicious bacilli by MALDI‐TOF MS confirmed the presence of *Rhodococcus erythropolis*. These species were further characterized as Gram‐positive bacteria (Figure 4).

It is noteworthy that the samples containing *R. erythropolis* initially tested positive for *M. ulcerans* after PCR analysis of lesion swabs collected from the wounds. Aliquots of lesion swabs (crude samples) were diluted to 1/100 in peptone water to reduce potential PCR inhibitors and some contaminating bacteria. Then, they were submitted to culture again, and the same results were obtained (yellowish growth colonies after 2 weeks). These data highlighted the persistence of *R. erythropolis* in the diluted samples and its presence in the crude samples collected directly from Buruli ulcer wounds. *M. ulcerans* may therefore coexist with *R. erythropolis* in Buruli ulcer patients. The data further reveal that *R. erythropolis* did not interfere in the detection specificity of *M. ulcerans* by IS2404/IPC multiplex qPCR. This is surely because of its low bacterial load in the crude samples or because of the high specificity of IS2404 oligonucleotides for *M. ulcerans*. In contrast, this specificity decreases during in vitro culture on LJ media where the fast‐growing *R*. *erythropolis* bacterium inhibited the growth of the slow‐growing *M. ulcerans* bacterium, complicating the biological diagnosis of *M. ulcerans* by in vitro culture.

Both Buruli ulcer lesions found with *R. erythropolis* were collected from a 34‐year‐old man and a 4‐year‐old child. These patients came from two different health districts (Edjom and Akonolinga urban) with an average distance of 40 km. The wounds were classified as new cases as they corresponded to the first Buruli ulcer–like lesions developed by these patients. The man had an active open sore well characteristic of Buruli ulcer disease with a circular Type III ulcer on the right lower limb. The ulcer at the granulation phase had a yellow‐to‐red background with edema of the foot (Figure 1). The man’s primary activity is fishing in the surrounding water bodies that have been classified as Buruli ulcer main risk factors in the area [9, 10]. The 4‐year‐old child at the prenursery level had a circular ulcer of the left upper limb at the granulation phase on articulation. The ulcer with a fibrinous background corresponds to a Type I ulcer. Both patients declared to have taken a treatment with rifampicin and clarithromycin for at least 4 weeks from the local health facility, but at the sample collection timepoint in the community, one of the patients (the 34‐year‐old man) was following a traditional therapy based on herbal decoctions and flowers in a local traditional healer house. All patients with a *M. ulcerans*–infected wound, including those described in this case‐report, were referred back to the nearest health facility or Akonolinga district hospital for proper management of the wound and treatment with a combination of rifampicin and clarithromycin for eight consecutive weeks following the WHO guidelines for management of Buruli ulcer cases.

*R. erythropolis* is a former *Mycobacterium* species that was originally called *Mycobacterium erythropolis* [3]. To strengthen our biochemical identification, a molecular confirmatory assay was attempted by WGS and sequence analyses to establish the genomic relatedness between this former *Mycobacterium* species and *M. ulcerans* and to generate baseline data that could help to characterize patients coinfected with both species. High molecular weight genomic DNA was extracted from pure culture yellowish colonies using the MagAttract HMW DNA kit (Qiagen, Germany). Sequencing libraries were prepared with a Nextera XT DNA Library Preparation Kit (Qiagen, Hilden, Germany) in a commercial company (Macrogen Europe, the Netherlands). Briefly, library clustering and WGS were performed on an Illumina HiSeq (paired‐end) platform with TruSeq Nano DNA (350) library preparation chemistry at Macrogen Europe. We sequenced three samples, including the culture suspensions from each of the two patients (AK003Y and AK007A) and one culture suspension (AK007B) resulting from a 1/100 dilution of sample AK007A. Short FASTQ reads from whole‐genome sequences were submitted to the NCBI‐BLAST*n* database, and the species was confirmed as *R. erythropolis*. Comparative genome sequence analysis of these samples with the *M. ulcerans* Agy99 reference genome [NC_008611.1] (chromosome only) revealed genome coverage of 5.67% (AK003Y), 5.73% (AK007A), and 5.79% (AK007B) (Table 1). High‐quality core‐genome data for *R. erythropolis* from sequenced samples were as high as 1.87 Gb on average. This was much larger than the 5.6 Mb of the *M. ulcerans* Agy99 reference genome size. The average genome coverage of 5.72% obtained by comparison of genome sequences of *R. erythropolis* and *M. ulcerans* Agy99 suggests very low genomic relatedness between both organisms. Whole‐genome sequences of *R. erythropolis* strains isolated from this study have been submitted to NCBI/SRA under BioProject PRJNA988007 to support further molecular characterizations and investigation of their biotechnology applications. The CARE checklist used to guide the reporting of this case report is provided as Supporting File 1.

**TABLE 1:** Genomic relatedness between whole‐genome sequences of *R. erythropolis* and *M. ulcerans* Agy99 (reference chromosome).

**Chromosome only**					
**Code**	**Genome size (Gb)**	**GC content (%)**	**Reference**	**Mapping (%)**	**Genome_coverage (%)**

AK003Y	1.66	60.34	*Mycobacterium ulcerans* Agy99, complete sequence [NC_008611.1]	3.20	5.67
AK007A	2.01	61.96		2.73	5.73
AK007B	1.95	61.72		2.63	5.79

## 3. Discussion

The study describes the first two cases of confirmed *Mycobacterium ulcerans*–infected lesions (Buruli ulcer cases) with *Rhodococcus erythropolis*. Species of the *Rhodococcus* genus have low pathogenicity and can cause diseases in animals, plants, and humans. Human infections caused by *Rhodococcus* species have mainly been associated with *R. equi,* and only limited cases have been reported for other species, including *R. rhodochrous*, *R. fascians* [13, 14], and *R. erythropolis* [4]. Immunocompromised patients constitute the most vulnerable group for infection by *Rhodococcus* bacterium. *R. erythropolis* is primarily found in soil. Human cases of this bacterium have been described in a conjunctival sample from a healthy human eye, in a patient’s bloodstream, and from sputum in a patient with pneumonia [4, 6]. Two additional cases have been reported in immunocompromised patients with HIV [7] and peritoneal dialysis [8]. No other studies have reported its presence on other human secretions or sites such as the skin surface. Most cases described so far have been found in unhealthy patients, which may highlight the increased public health interest in this pathogen infecting humans and causing disease. In our case, it may be considered a secondary bacterium that takes advantage of primary infections to invade human secretions and open surfaces such as Buruli ulcer wounds. In culture, *R. erythropolis* shows rough and orange to red colonies after coloration with glucose yeast extract agar and Sauton’s agar [1]. However, this fast‐growing bacterium revealed a yellowish phenotype on LJ media 14 days postincubation at 33°C. These colonies appeared to be closely related to typical *M. ulcerans* colonies on LJ media. This phenotypic relatedness may result in false‐positive diagnoses of *M. ulcerans* during routine laboratory culture procedures, and it may also affect the efficiency of phenotypic tests based on cultured colonies and molecular assays such as WGS that rely on pure culture isolates of a given organism. Further analyses by ZN staining revealed closely related phenotypes for both species under the microscope. This suggests that microscopic analysis by ZN staining is not sufficient to clearly differentiate the two species. However, AFB of *R. erythropolis* did not appear in a cluster compared to the AFB of *M. ulcerans*, which usually appears in a cluster or clump with the possibility of forming cords or branching filaments after ZN staining. MALDI‐TOF MS analyses have shown high resolution in differentiating *M. ulcerans* to *R. erythropolis.* Although this technique is costly and not always available in many resource‐limited laboratories, it can be used as a confirmatory assay to characterize *M. ulcerans* colonies in Buruli ulcer endemic areas.

Contamination of *M. ulcerans*–infected lesions by *R. erythropolis* has never been described. Thus, our study represents the first report of skin lesion contamination with this organism. Only a few coinfected cases of *M. ulcerans* with cutaneous leishmaniasis, HIV, *E. coli*, and several secondary microbial pathogens (*Staphylococcus aureus, Bacillus* spp.*, Proteus* spp., *Pseudomonas aeruginosa*, *Streptococcus* spp., and *Candida albicans*) have been reported [15–18]. Although HIV and cutaneous leishmaniasis are associated with specific disease symptoms in coinfected individuals, skin colonization by *R. erythropolis* did not reveal any visual abnormal features in Buruli ulcer lesions. Nevertheless, the source of contamination of Buruli ulcer lesions by this organism remains elusive. Considering the routine activities of infected individuals (fishing, bathing in rivers, and drinking river water), we suspect direct contamination from the soil or other environmental sources of *R. erythropolis* or indirect contamination from plant materials used by local traditional healers to protect and treat Buruli ulcer wounds in this area. The analyses confirmed the viability of *R. erythropolis* found in the wounds, and this contamination may have been eased by the open sore caused by *M. ulcerans*. Nevertheless, its clinical impact on Buruli ulcer disease progression and treatment outcomes has not been investigated here. Previous coinfection cases with HIV can complicate the management of the patient, making Buruli ulcer clinical progression more aggressive and resulting in poor treatment outcomes [19]. Such investigations are needed for Buruli ulcer patients carrying viable germs of *R. erythropolis* to facilitate wound management control and the timely treatment of the disease. Moreover, this study suggests the necessity to investigate the microbiota of all skin injuries found in Buruli ulcer endemic localities for a better understanding of pathogens causing non‐Buruli ulcer wounds.

The data from this study highlight the necessity to perform confirmatory assays of clinical *M. ulcerans* culture isolates by PCR and microscopy prior to downstream analyses. Consequently, archived Buruli ulcer samples that were previously classified as negative could be reevaluated using qPCR‐based methods to provide a more accurate assessment of disease burden within affected communities. This also applies to environmental *M. ulcerans* culture isolates where the risk of contamination by *R. erythropolis* and other fast‐growing bacteria may be more significant due to its everlasting presence in the soil and environment. Analysis of whole‐genome sequences further showed that although *M. ulcerans* and *R. erythropolis* may share similar phenotypes during in vitro culture on LJ media and ZN staining, they are unlikely to be related at the genomic level. This highlights the importance of molecular tools for pathogen genomic surveillance and the characterization of infectious organisms, especially in the present era of the Global Health concept of the World Health Organization.

## 4. Conclusion

We report two contamination cases of *M. ulcerans*–infected lesions with *R. erythropolis* in Buruli ulcer patients. Differential microbial analysis of crude lesion samples from these patients revealed that *R. erythropolis* may not interfere in the detection specificity of *M. ulcerans* by IS2404/IPC‐qPCR multiplex analysis. However, this former *Mycobacterium* species can likely inhibit *M. ulcerans* growth in recommended LJ culture media, complicating routine biological diagnosis by culture and microscopy. Hence, Buruli ulcer is likely to be underdiagnosed in lesions colonized with *R. erythropolis.* We further provided evidence that the genome sequence of *R. erythropolis* is less related to the *M. ulcerans* genome, supporting its classification in the *Rhodococcus* genus.

## Author Contributions

Conceptualization: Francis Zeukeng, Anthony Ablordey, Jude Daiga Bigoga, and Solange E. Kakou‐Ngazoa; investigation: Francis Zeukeng, Anthony Ablordey, Jude Daiga Bigoga, Evelyne Fegue Ndzodo, Solange E. Kakou‐Ngazoa, and David N’golo Coulibaly; formal analysis: Francis Zeukeng, Jennifer Seyram Amedior, and Anthony Ablordey; data curation: Francis Zeukeng, Jennifer Seyram Amedior, and David N’golo Coulibaly; sequence submission to public repositories: Francis Zeukeng; funding acquisition and resources: Francis Zeukeng, Jude Daiga Bigoga, and Anthony Ablordey; supervision and administration: Anthony Ablordey, Jude Daiga Bigoga, Stephen Mbigha Ghogomu, Solange E. Kakou‐Ngazoa, and Wilfred Fon Mbacham; writing–original draft: Francis Zeukeng and Jennifer Seyram Amedior; and writing–review and editing: David N’golo Coulibaly, Anthony Ablordey, Jude Daiga Bigoga, Stephen Mbigha Ghogomu, Solange E. Kakou‐Ngazoa, and Wilfred Fon Mbacham.

## Funding

This study was funded by the EDCTP2 program supported by the European Union (grant number: TMA2019PF‐2693‐AGBBU).

## Disclosure

The funders had no role in the study design, data collection, analysis, decision to publish, or manuscript preparation. Initial research findings from this study were presented at a conference [20], and this manuscript presents and discusses an extended version of the published conference abstract. All authors have read and approved the final manuscript.

## Ethics Statement

Approval to conduct the study was obtained from the Cameroon national ethics review committee (No. 946/CE/CNERSH/SP).

## Consent

Written informed consent was obtained from the patient and legal guardians for publication of this case report and any accompanying images. A copy of the written consent is available for review by the Editor‐in‐Chief of this journal.

## Conflicts of Interest

The authors declare no conflicts of interest.

## Supporting Information

Additional supporting information can be found online in the Supporting Information section.

## Supporting information


**Supporting Information** The CARE checklist (CARE‐checklist‐English) used to guide the reporting of this case report is provided as Supporting File 1.

## Data Availability

Sequence data that support the findings of this study have been deposited in the NCBI/SRA public repository with the BioProject accession number PRJNA988007.
